# Do differentiated service delivery models for HIV treatment in sub-Saharan Africa save money? Synthesis of evidence from field studies conducted in sub-Saharan Africa in 2017-2019

**DOI:** 10.12688/gatesopenres.13458.1

**Published:** 2021-12-08

**Authors:** Sydney Rosen, Brooke Nichols, Teresa Guthrie, Mariet Benade, Salome Kuchukhidze, Lawrence Long

**Affiliations:** 1 Department of Global Health, Boston University School of Public Health, Boston, MA, 02118, USA; 2 Health Economics and Epidemiology Research Office, University of the Witwatersrand Faculty of Health Sciences, Johannesburg, 2193, South Africa

**Keywords:** Antiretroviral therapy; HIV; Africa; costs; differentiated service delivery

## Abstract

**Introduction**: “Differentiated service delivery” (DSD) for antiretroviral therapy (ART) for HIV is rapidly being scaled up throughout sub-Saharan Africa, but only recently have data become available on the costs of DSD models to providers and patients. We synthesized recent studies of DSD model costs in five African countries.

**Methods**: The studies included cluster randomized trials in Lesotho, Malawi, Zambia, and Zimbabwe and observational studies in Uganda and Zambia. For 3-5 models per country, studies collected patient-level data on clinical outcomes and provider costs for 12 months, and some studies surveyed patients about costs they incurred. We compared costs of differentiated models to those of conventional care and identified drivers of cost differences. We also report patient costs of seeking care.

**Results**: The studies described 22 models, including facility-based conventional care. Of these, 13 were facility-based and 9 community-based models; 15 were individual and 7 group models. Average provider cost/patient/year ranged from $100 in Zambia to $187 in Zimbabwe, in both cases for facility-based conventional care. Conventional care was less expensive than any other model in the Zambia observational study, more expensive than any other model in Lesotho, Malawi, and Zimbabwe, and in the middle of the range in the Zambia trial and the observational study in Uganda. Models incorporating 6-month dispensing were consistently less expensive to the provider per patient treated. Savings to patients were substantial for most models, with patients’ costs roughly halved.

**Conclusion**: In five field studies of the costs of DSD models for HIV treatment, most models within each country had relatively similar costs, except for 6-month dispensing models, which were slightly less expensive. Most models provided substantial savings to patients. Research is needed to understand the effect of DSD models on the costs of ART programmes as a whole.

## Introduction

Throughout sub-Saharan Africa, governments are rapidly scaling up “differentiated service delivery” (DSD) for antiretroviral therapy (ART) for HIV. DSD tailors the location, frequency, and other characteristics of treatment delivery to specific patient populations, with the goal of making treatment both more patient-centric and more efficient for service providers ^1^. DSD is intended to improve the “conventional” model of service delivery, in which all ART patients received the same, resource-intensive, clinic-based care regardless of their conditions, constraints, or preferences. Examples of DSD models for ART delivery include medication pickup points at convenient locations in the community, multi-month dispensing of medications that minimize the frequency of required clinic visits, and adherence clubs that bring groups of patients together either at a healthcare facility or in the community for medication refills and social support.

Among other anticipated benefits, DSD models are expected to reduce the cost of service delivery per ART patient for providers and the cost of accessing ART for patients themselves ^2^. This expectation follows logically on the notion that DSD models are designed to be “less intensive” than conventional care, and therefore presumably utilize fewer resources per patient served ^3^. Until very recently, however, few empirical data drawn from DSD models implemented in routine care settings have been available to compare the costs of differentiated service delivery to conventional, facility-based care ^4^.

Between 2017 and 2019, we conducted or participated in five studies of DSD model costs and patient outcomes in sub-Saharan Africa, in Lesotho, Malawi, Uganda, Zambia, and Zimbabwe. All were sponsored by the U.S. Agency for International Development and the U.S. President’s Emergency Program for AIDS Relief (PEPFAR) under the EQUIP Health project. Clinical outcomes and costs of these studies have previously been reported ^5– 11^. All utilized primary healthcare clinics and/or hospital outpatient clinics to implement and evaluate between three and six differentiated models of delivery of antiretroviral therapy for stable, adult patients. All estimated provider costs; several also estimated costs to recipients of treatment. Each reported, for each DSD model in the study, the proportion of patients achieving a primary outcome of retention in care 12 months after study enrollment, an average cost per patient enrolled, an average cost per patient retained or suppressed, and, for those that included patient costs, an average cost incurred by each patient per year of care. In this paper, we aggregate and synthesize these data to provide a larger picture of the resource utilization and costs of DSD models in the sub-Saharan region.

## Methods

In each country, an analysis was conducted of the outcomes and costs of DSD models that were mandated in national guidelines and/or introduced by nongovernmental treatment partners in collaboration with local ministries of health. Three of the analyses were cluster-randomized trials; the remaining two were observational cohort studies of routine care. One of the cluster-randomized trials was conducted in two countries and reported costs by country; we include each country separately here, giving us a total of six sets of country-level results for our analysis.

In Table 1, we describe each of the study sites, designs, populations, and costing methods. The five studies utilized similar but not identical methods. Methods are described in detail in the previous reports for each study, cited above. Each analysis collected individual patient-level data on the clinical outcomes of patients enrolled in DSD models for at least 12 months and the costs of the resources used to treat the patients during that period. In each country, the analysis included conventional, clinic-based care as one of the study models. Most models enrolled only “stable” adult patients, typically ≥18 years old, with at least six months experience on ART and with evidence of a suppressed viral load, except where indicated in Table 1. For all studies except that in Uganda, the period of observation corresponded with patients’ first 12 months enrolled in a DSD model; in Uganda, study participants had already participated in the models for a median of one year at study enrollment, and we report results for months 13–24 after study enrollment. We note that each of the papers included in this synthesis contains some stratification of results by patient and/or facility characteristics which we do not present here.

**Table 1.** Studies included in the analysis.

Country and sources	Study design	Population enrolled	Period of enrollment and follow up or observation	Definition of retention in care	Costs included	Costs excluded	Patient costs captured?
Lesotho ^6, 11^	Prospective cluster randomized trial at 30 healthcare facilities	Adults on first line ART ≥6 months with suppressed viral load	Aug 2017- July 2019; first 12 months of model participation for each patient	Not having missed a scheduled clinic visit or DSD interaction for >90 consecutive days	ARVs, viral load tests, clinic visits and off-site DSD model interactions, infrastructure, equipment, transport	Non-ARV medications, other laboratory tests, above-site costs	Yes
Malawi (INTERVAL) ^5^	Prospective cluster randomized trial at 15 healthcare facilities	Adults on first line ART ≥ 6 months with suppressed viral load	May 15, 2017-April 30, 2018; first 12 months of model participation for each patient	No period of >60 days without possession of ARVs, based on dates and quantities dispensed	ARVs, outpatient clinic visits that included ARV refills, and infrastructure	Laboratory tests, clinic visits that did not include an ARV refill, above-site costs	Yes
Uganda ^8^	Observational cohort using retrospective, routinely collected medical record data from 20 healthcare facilities, many of which offered multiple models of care	Adults on first or second line ART; included new, non- suppressed, and advanced disease patients in some models	Jan 1, 2017-Dec 31, 2018; 24 months follow up for all patients (outcomes for months 13-24 reported here)	Not having missed a scheduled clinic visit or DSD interaction for >90 consecutive days; this study also reported viral suppression at 12 months	ARVs and other medications (with supply chain costs), laboratory tests, clinic visits and off-site DSD model interactions, infrastructure, equipment, transport, training, administration, above- site costs		No
Zambia1 ^7^	Observational cohort using retrospective, routinely collected medical record data from 20 healthcare facilities	Adults on first line ART ≥6 months with suppressed viral load (excluding mobile ART model); many model enrollees did not have a record of a suppressed viral load	Jan 1, 2015-Dec 31, 2017; first 12 months of model participation for each patient	Having a recorded clinic visit between 9 and 15 months after enrollment in the DSD model (all models required a minimum of one visit every 12 months)	ARVs and other medications, laboratory tests (except for mobile model), clinic visits and off-site DSD model interactions, infrastructure, equipment, transport	Above-site costs; lab costs excluded for mobile ART model	No
Zambia2 (INTERVAL) ^5^	Prospective cluster randomized trial at 15 healthcare facilities	Adults on first line ART ≥ 6 months with suppressed viral load	May 15, 2017-April 30, 2018; first 12 months of model participation for each patient	No period of >60 days without possession of ARVs, based on dates and quantities dispensed	ARVs, outpatient clinic visits that included ARV refills, and infrastructure	Laboratory tests, clinic visits that did not include an ARV refill, above-site costs	Yes
Zimbabwe ^9, 10^	Prospective cluster randomized trial at 30 healthcare facilities	Adults on first line ART ≥6 months with suppressed viral load	Aug 2017-Feb 2018; first 12 months of model participation for each patient	Not having missed a scheduled clinic visit or DSD interaction for >90 consecutive days	ARVs, clinic visits and off-site DSD model interactions, viral load tests	Other laboratory tests, above-site costs	Yes

In addition to the average cost per patient enrolled, each study also reported the average cost per patient achieving a primary outcome of retention 12 months after study enrollment. Definitions of retention varied somewhat among the studies and are included in Table 1.

For this synthesis, we collated provider cost results per patient for each model of care from each country, with resources and costs broken down into six categories: ARV medications, non-ARV medications, laboratory tests, facility visits, DSD-model interactions distinct from full facility visits, and infrastructure and fixed costs. Most of the studies included some but not all of these categories. DSD interactions included all meetings of providers and patients that occurred away from the clinic, group meetings of patients both at and away from clinics, and individual facility visits that were designed specifically for a differentiated model, such as a “fast track visit”. In some studies, costs for infrastructure and other fixed resources were allocated per visit and included in the estimated cost/visit, rather than as a separate category, and we note where this occurs.

We note major exclusions from cost/patient estimates for each study. We also compare observed, average numbers of clinic visits and DSD interactions completed by each patient with the numbers recommended in each model’s guidelines. Finally, we report patient costs per year in care, divided into out-of-pocket (cash) costs, such as transport, and opportunity costs, valued at the country’s minimum wage for the time required per healthcare system interaction.

No ethics review was required for this synthesis analysis, as all data used were previously published in the sources cited.

## Results

### Models and study populations

The five studies included patient outcomes and cost results for 22 models of care, including conventional models in each country. In Table 2, we list the individual DSD models included in the studies in each country, the numbers of participants observed (sample sizes), and the proportion achieving the common outcome of retention in care at 12 months and compare the models based on their location of services, duration of dispensing, and group or individual approach. We note that, with the exception of Uganda and the conventional care models, all the models in the studies were designed and implemented by external parties—either nongovernmental organizations or researchers—and not by the healthcare systems themselves. In Uganda, in contrast, all models evaluated were part of the national DSD strategy.

**Table 2.** Description of models and sample included in each study.

Model name	Description of model	Number of model participants in study	% retained at 12 months	Location		Refill duration			Approach	
				Facility	Community	1-2 mos	3 mos	6 mos	Individual	Group
**Lesotho**										
Facility enhanced conventional care ^††^	Standard facility-based care with 4 visits/year and 3-month refills	1,898	97.1%	✓			✓		✓	
Community ART groups with 3-month refills (CAGs)	Group of 6-12 patients in same geographic area; groups meet 4 times/year in community, with 1 member collecting medications for all members from clinic once/quarter and 3-month refills	1,558	96.5%		✓		✓			✓
Community distribution points with 6-month refills	6-month dispensing alternating between clinic and community pickup point; 1 clinic visit + 1 pickup point visit/year	1,880	94.7%		✓			✓	✓	
**Malawi**										
Facility conventional care	Standard of care; dispensing intervals varied with provider’s discretion, availability of stock, etc. from 1-3 months, with 4-12 clinic visits/year.	1,532	89.7%	✓						
Facility dispensing with 3-month refills	Patients consistently received 3-month supplies of ARVs, with 4 clinic visits/year. No other changes to model of care.	1,430	90.2%	✓			✓		✓	
Facility dispensing with 6-month refills	Patients consistently received 6-month supplies of ARVs, with 2 clinic visits/year. No other changes to model of care.	1,588	93.2%	✓				✓	✓	
Uganda *										
Facility conventional care	Standard of care referred to as “Facility- based individual model”; generally required 4 clinic visits/year with varying dispensing intervals. Note: This was considered a differentiated model for new and complicated patients but continued to function as conventional care for patients not in other models	128	97% (88% *)	✓		✓			✓	
Facility-based groups	Groups of patients requiring additional care or adherence support, with varying numbers of visits and dispensing intervals. **	129	96% (94%)	✓		✓				✓
Fast-track drug refills	Accelerated medication pickup at facilities for stable first- and second-line patients; varying dispensing intervals.	133	99% (90%)	✓		✓	✓		✓	
Client-led ART delivery (CAGs)	Groups of stable patients meet in the community, with 1 member collecting medications from the clinic for all members; varying frequency of meetings and dispensing intervals.	131	98% (90%)		✓	✓	✓			✓
Community drug distribution points	Stable patients pick up medications from a community location, including private pharmacies; dispensing intervals varied but most patients had 1 clinic visit and 6 medication pickup interactions per year.	132	100% (92%)		✓	✓			✓	
**Zambia 1 (observational)**										
Facility conventional care	Standard of care; generally requires 4 full clinic visits/year with 1-3 month dispensing intervals. For study, selected a matched sample of DSD model-eligible patients not enrolled in DSD models.	1,174	80.7%	✓		✓			✓	
Community adherence groups (CAGs)	Group of approximately 6 patients meet monthly in the community; 1 member collects medications for all members. 2 full clinic visits/year.	754	83.2%		✓	✓				✓
Urban adherence groups	Group of 20-30 patients meet as a group at clinic to receive services every 2-3 months. Urban areas only.	193	94.8%	✓		✓				✓
Home ART delivery	Community health workers visit patients’ homes to deliver medications and monitor treatment. 2 full clinic visits/year. Rural areas only.	169	79.3%		✓	✓			✓	
Mobile ART services ^†^	Clinical team from district hospital visits rural health posts every 2 weeks to provide services. Requires 6 patient interactions/ year.	216	68.5%		✓	✓			✓	
**Zambia 2**										
Facility conventional care	Standard of care; dispensing intervals vary with provider’s discretion, availability of stock, etc. from 1-3 months, with 4-12 clinic visits/year.	1,480	74.6%	✓		✓			✓	
Facility dispensing with 3-month refills	Patients consistently receive 3-month supplies of ARVs, with 4 clinic visits/year. No other changes to model of care.	1,296	82.3%	✓			✓		✓	
Facility dispensing with 6-month refills	Patients consistently receive 6-month supplies of ARVs, with 2 clinic visits/year. No other changes to model of care.	1,393	89.7%	✓				✓	✓	
**Zimbabwe**										
Facility enhanced conventional care ^††^	Standard facility-based care with 4 clinic visits/year and 3-month refills	1,919	93.0%	✓			✓		✓	
Community ART groups with 3-month refills	Group of 6–12 patients in same geographic area; groups met 4 times/year in community, with 1 member collecting medications for all members from clinic once/quarter and 3- month refills; 1 annual clinical consultation/ year on same day for entire group.	1,335	94.8%		✓		✓			✓
Community ART groups with 6-month refills	Group of 6–12 patients in same geographic area; groups met 4 times/year in community, with 1 member collecting medications for all members from clinic once/quarter and 6- month refills; 1 annual clinical consultation/ year on same day for entire group.	1,546	95.5%		✓			✓		✓

*Uganda results reported were from the second 12-month observation period (months 13–24 reported in published paper). Outcome in parentheses is viral suppression.

** Facility groups included in study were for pregnant and post-partum women only.

†Model includes newly-initiated patients; outcomes reflect high early attrition during period when patients are not eligible for all other DSD models.

††Conventional models in Lesotho and Zimbabwe were an enhanced version of standard of care in which providers were asked to dispense 3-month supplies of ARVs, rather than whatever duration they otherwise would have and patients received 4 clinical consultations per year, rather than 1.

### Provider cost per patient

The average cost per patient included in the analysis and per patient retained at the 12-month endpoint, by country and model, is shown in Table 3, with the breakdown into cost categories in Table 4. We include the 95% confidence interval or standard deviation for each cost estimate as provided by the original publications.

**Table 3.** Average annual cost and cost per patient retained at 12 months, by country and model, in USD.

Country and model	Mean annual cost per patient (SD or 95% CI where reported)	Mean annual cost per patient retained for 12 months (SD or 95% CI where reported)	% difference from SOC model (mean annual cost per patient)
**Lesotho (2018 USD)**			
Facility care with 3-month refills (SOC)	$122.28 (23.91)	$125.99 (24.64)	
Community ART groups with 3-month refills (CAG)	$114.20 (23.03)	$118.38 (23.87)	-6.6%
Community distribution points with 6-month refills	$112.58 (21.44)	$118.83 (22.63)	-7.9%
Malawi (2018 USD) *			
Facility conventional care (SOC)	$86.50 (84.50-88.42) *	$96.15	
Facility dispensing with 3-month refills	$86.00 (83.99-87.91) *	$94.87	-0.6%
Facility dispensing with 6-month refills	$84.60 (82.62-86.54) *	$90.76	-2.2%
**Uganda (2018 USD)**			
Facility-based individual management (SOC)	$152.49 (72.04)	$173	
Facility groups (pregnant/post-partum)	$141.29 (33.70)	$150	-7.3%
Fast-track drug refills	$166.48 (82.51)	$185	+9.2%
Client-led ART delivery (CAG)	$150.07 (54.94)	$167	-1.5%
Community drug distribution points	$146.42 (59.52)	$159	-3.9%
**Zambia1 (2018 USD)**			
Facility conventional care (SOC)	$100.09 (61.59)	$124	
Community adherence groups (CAG) ^‡^	$116.25 (67.83)	$140	+16.1%
Urban adherence groups ^‡^	$147.01 (57.15)	$155	+46.9%
Mobile ART services *	$122.46 (70.10)	$179	+22.3%
Home ART delivery ^‡^	$137.18 (57.02)	$173	+37.1%
Zambia2 (2018 USD) *			
Facility conventional care (SOC)	$132.00 (130.43-134.35) *	$177.00	
Facility dispensing with 3-month refills	$134.00 (132.09-136.02) *	$162.87	+1.5%
Facility dispensing with 6-month refills	$128.00 (125.64-129.57) *	$142.41	-3.0%
**Zimbabwe (2020 USD)**			
Facility dispensing with 3-month refills (SOC)	$187.04 (185.31-188.78)	$195.06 (194.11-195.99)	
Community ART groups with 3-month refills	$177.83 (176.19-179.46)	$182.81 (181.80-183.83)	-6.3%
Community ART groups with 6-month refills	$167.40 (165.44; 169.36)	$172.81 (171.30; 174.31)	-11.4%

*Excludes cost of laboratory tests, for which the authors did not have data; one viral load test per year, as called for by national guidelines, would add approximately $19 to the mean annual cost per patient per year. ^‡^Table shows results from lower cost scenario reported by source publication.

**Table 4.** Average number of healthcare system interactions per patient per year, guideline and observed, and average ARV dispensing duration.

Country and model	Clinic visits/year		DSD interactions/year	
	Guidelines ^†^	Observed	Guidelines	Observed
**Lesotho**				
Facility care with 3-month refills (SOC)	4	4.19	0	0.00
Community ART groups with 3-month refills	1	1.00 *	4	4.65
Community distribution points with 6-month refills	1	1.49	1	0.96
**Malawi**				
Facility conventional care (SOC)	4-12	5.4	0	0.00
Facility dispensing with 3-month refills	3	4.9	0	0.00
Facility dispensing with 6-month refills	2	2.9	0	0.00
**Uganda**				
Facility-based individual management (SOC)	4-12	7.63	0	0.00
Facility groups (pregnant/post-partum)	2	9.05	2-4	6.6
Fast-track drug refills	4	5.82	0	0.00
Client-led ART delivery	2	5.92	2	2.00
Community distribution points	1	6.07	4	1.92
**Zambia 1**				
Facility conventional care (SOC)	4	2.55	0	0.00
Community adherence groups	2	2.64	12	10.02
Urban adherence groups	2	3.06	4	4.54
Mobile ART services	0	0.00	6	4.87
Home ART delivery	1	1.01	6	3.34
**Zambia 2**				
Facility conventional care (SOC)	4-12	4.6	0	0.00
Facility dispensing with 3-month refills	3	4.7	0	0.00
Facility dispensing with 6-month refills	2	2.8	0	0.00
**Zimbabwe (2020 USD)**				
Facility dispensing with 3-month refills (SOC)	4	5.02	0	0.00
Community ART groups with 3-month refills	1	1.99 *	4	3.05
Community ART groups with 6-month refills	1	1.98 *	2	1.18

*Assumed based on source authors’ calculations. †Patients are always permitted to make additional clinic visits as needed; guidelines should be regarded as the minimum number of clinic visits/year.

If we assume that patients in Malawi had an average of one viral load test per year, adding roughly $19 to the cost/patient year, then average costs/patient for the 12-month observation period ranged from a low of $100/patient for conventional, facility-based care in Zambia to a high of $187 for conventional, facility-based care in Zimbabwe. Importantly, we note that these values are not adjusted to reflect differences in purchasing power across countries. Each country pays different procurement prices for commodities such as medications and has different salary scales (Table S1). As a result, differences in cost/patient between countries are generally larger than the differences among models within countries, and comparisons between countries are less informative than comparisons within countries. We focus on cost differences between models within countries for the remainder of this paper, while also comparing resource utilization across countries where possible.

Conventional, facility-based care was less expensive than any other model in the observational study in Zambia, more expensive than any other model in the trials in Lesotho, Malawi, and Zimbabwe, and in the middle of the range for the trial in Zambia and the observational study in Uganda. Models incorporating six-month dispensing were consistently the least expensive per patient treated. In most of the countries, most models cost slightly less per patient than did conventional care. The exception to this is Zambia1, where all the DSD models were estimated to cost more than SOC. Cost differences between the least and most expensive models within each country were smaller for the cluster-randomized trials (2–14%) but more substantial for the two observational studies (15% in Uganda and 32% in Zambia). This was in part because the observational studies, which reflect use of DSD models in routine care, included varying numbers of patients on expensive second line therapy and/or in their first 6 months on ART, a period that tends to be resource-intensive; healthcare system interactions per patient also diverged from guidelines more in the observational studies than in the trials, as reported below.

### Allocation of costs to cost categories

Reasons for cost differences between models in the same countries become apparent in Table 4 and Table 5, which provide a breakdown of average utilization of healthcare system interactions and of costs per patient by resource category.

**Table 5.** Breakdown of cost per patient at 12 months, by model (cost and percentage of total mean cost/patient).

Country and model	ARVs		Non-ARV medications		Lab tests		Clinic visits		DSD interactions		Infrastructure and fixed costs	
**Lesotho**												
Facility care with 3-month refills (SOC)	$84.37	69%	^*^		$12.00	10%	$25.91	21%	$0	0%	^†^	
Community ART groups with 3-month refills	$86.10	75%	^*^		$8.93	8%	$5.78	5%	$13.59	12%	^†^	
Community distribution points with 6- month refills	$87.08	77%	^*^		$10.29	9%	$9.60	9%	$5.59	5%	^†^	
**Malawi** ¶												
Facility conventional care (SOC)	$77.54	87%	^*^		^‡^		$8.19	9%	$0	0%	$3.32	4%
Facility dispensing with 3-month refills	$77.65	88%	^*^		^‡^		$7.43	8%	$0	0%	$3.32	4%
Facility dispensing with 6-month refills	$78.18	91%	^*^		^‡^		$4.42	5%	$0	0%	$3.32	4%
**Uganda** ^**^												
Facility-based individual management (SOC)	$115.33	76%	$9.99	7%	$13.04	9%	$5.00	3%	$0.00	0%	$9.12	6%
Facility groups (pregnant/post-partum)	$96.88	69%	$13.13	9%	$14.85	11%	$6.90	5%	$0.06	0%	$9.46	7%
Fast-track drug refills	$133.96	80%	$11.10	7%	$11.75	7%	$4.77	3%	$0.00	0%	$4.89	3%
Client-led ART delivery	$103.20	69%	$20.10	13%	$11.21	7%	$2.55	2%	$0.17	0%	$12.84	9%
Community distribution points	$112.76	77%	$10.12	7%	$11.40	8%	$1.47	1%	$0.17	0%	$10.51	7%
**Zambia1** §												
Facility conventional care (SOC)	$86.04	86%	$0.13	0%	$4.61	5%	9.31	9%	$0	0%	^†^	
Community adherence groups	$89.01	77%	$0.10	0%	$6.92	6%	$9.63	8%	$9.92	9%	^†^	
Urban adherence groups	$101.87	69%	$0.18	0%	$23.24	16%	$11.16	8%	$10.68	7%	^†^	
Mobile ART services	$73.30	60%	$3.45	3%	^‡^		$0.00	0%	$45.71	37%	^†^	
Home ART delivery	$87.96	64%	$0.18	0%	$4.56	3%	$3.70	3%	$40.78	30%	^†^	
**Zambia2** ¶												
Facility conventional care (SOC)	$109.65	76%	^*^		^‡^		$31.75	22%	$0	0%	$2.20	2%
Facility dispensing with 3-month refills	$106.71	75%	^*^		^‡^		$32.69	23%	$0	0%	$2.20	2%
Facility dispensing with 6-month refills	$109.45	83%	^*^		^‡^		$19.48	15%	$0	0%	$2.20	2%
**Zimbabwe** ¶												
Facility dispensing with 3-month refills (SOC)	$164.17	84%	^*^		$6.90	4%	$23.98	12%	$0	0%	^†^	
Community ART groups with 3-month refills	$163.45	89%	^*^		$6.43	4%	$9.23	5%	$3.70	2%	^†^	
Community ART groups with 6-month refills	$161.08	93%	^*^		$1.08 ^§§^	1%	$9.22	5%	$1.42	1%	^†^	

*Non-ARV medication costs not captured. †Infrastructure and other fixed costs included in clinic visit costs. ‡Laboratory costs not captured; one viral load test per year, as called for by national guidelines, would add approximately $19 to the mean annual cost per patient per year. §For Zambia1, on-site pharmacy costs were included in clinic visit costs. ¶Costs for Malawi, Zambia2, and Zimbabwe are only for those who were retained at 12 months. **For Uganda, 1) medication costs include supply chain costs, not solely procurement of products; 2) lab tests included tests other than viral loads; and 3) infrastructure and fixed costs included some costs incurred by implementing partners above the level of the individual healthcare facility. ^§§^In Zimbabwe, facilities in the study arm with community ART groups with 6 month refills were more likely to be located in districts that did not have access to viral load testing technology. As a result, far fewer patients received viral load tests than in the other arms ( Table 4), and average cost per patient was low.

In the cluster randomized trials (Lesotho, Malawi, Zambia2, Zimbabwe), utilization of facility visits and DSD interactions was roughly consistent with guidelines for each DSD model, though even under trial conditions, patients were likely to interact with the healthcare system (either through facility visits or DSD interactions) more often than guidelines recommended. Most of the DSD models reduced the number of clinic visits/patient/year substantially compared to conventional care, from at least four in conventional care to three, two, or even one per year in the DSD models. Some of the models were designed to replace multiple clinic visits with an even larger number of DSD interactions; others reduced the overall burden of healthcare system interactions faced by most patients. In general, patient interaction with the healthcare system was minimized in six-month dispensing models and was relatively frequent in group models.

In the observational studies (Uganda and Zambia1), average numbers of facility visits and DSD interactions diverged somewhat more widely from guideline recommendations, with some models experiencing more and others fewer healthcare system interactions than expected. This variation appears to reflect a combination of patients’ choices, providers’ preferences, and model design. Patients are always permitted to make more clinic visits than suggested by guidelines, based on individual needs; in some cases, extra interactions likely indicate shorter dispensing intervals, forcing patients to return more often for medication refills. Fewer interactions may suggest that patients are experiencing lapses in medication adherence or may, in contrast, reflect longer dispensing intervals. Six-month dispensing models are designed to require only 2 interactions per year, but all of the studies in Table 4 showed an average of 2.5–3.0 interactions (combined facility visits + DSD interactions) per year, indicating that patients interacted with the healthcare system more often than model designers intended and making these models slightly more expensive than was likely anticipated.

The differences among models within countries for ARV costs per patient largely reflect loss to follow up--patients who did not receive 12 months of medications cost less and brought the average down—and differing proportions of patients on second-line regimens, which are more expensive than first-line regimens. In most models, patients received <1 viral load test per year; the average quantity of viral load tests utilized per patient per year ranged from 0.22 to 1.15. Costs of clinic visits and DSD interactions varied widely, as would be expected, since this is the characteristic of treatment that DSD models change most (though it is also a relatively inexpensive resource).

One of the major differences among DSD models for HIV treatment is dispensing interval (number of months of ARV medications dispensed at a time). All of the cluster randomized trials specified dispensing interval as a characteristic of the model. Actual dispensing intervals are reported for only two studies. In the INTERVAL trial (Malawi and Zambia2), participants received the expected supply of ARVs roughly 90% of the time; some patients in the 3-month dispensing arm received more or fewer than 3 months’ supply at some or all visits, and some patients in the 6-month dispensing arm received fewer than 6 months at some or all visits. In Uganda, the average dispensing interval ranged from 1.3 to 2.1 months per medication pickup, helping to explain the relatively high number of healthcare system interactions observed for the models in Uganda.

### Patient costs

Table 6 presents estimates of costs borne by patients for the studies that included a patient survey. Total costs/patient/year depend heavily on how individuals’ time is valued and the estimated or assumed duration of an average clinic visit or DSD interaction, but in general, savings to patients were substantial for almost all of the differentiated models, equivalent to several days’ minimum wage in each country. All of the studies in Table 6 compared three- and six-month dispensing to conventional care, either without making any other changes (Malawi and Zambia2) or using community-based models (Lesotho and Zimbabwe). Patient savings were large for all the six-month models, while savings for the three-month models likely depended on the visit frequency required by conventional care, relative to the quarterly interaction frequency required by three-month dispensing.

**Table 6.** Average costs to patients per year in care, by model.

Country and model	Transport	Opportunity cost	Total cost	% difference from SOC model (mean total cost per patient)	Minimum wage reported for country ^‡^
**Lesotho**					
Facility care with 3-month refills	$11.45	$32.97 *	$44.42		$7.10
Community ART groups with 3-month refills	$2.62	$13.73 *	$16.34	-63.2%	
Community distribution points with 6-month refills	$4.83	$13.94 *	$18.77	-57.7%	
**Malawi**					
Facility conventional care	$1.59 §	$5.30 ^†^	$6.89		$1.33
Facility dispensing with 3-month refills	$1.59 §	$6.63 ^†^	$8.22	+19.3%	
Facility dispensing with 6-month refills	$2.27 §	$3.98 ^†^	$6.25	-9.3%	
**Zambia 2**					
Facility conventional care	$1.67 §	$15.00 ^†^	$16.67		$4.99
Facility dispensing with 3-month refills	$1.58 §	$20.00 ^†^	$21.58	+29.5%	
Facility dispensing with 6-month refills	$1.19 §	$9.98 ^†^	$11.17	-33.0%	
**Zimbabwe**					
Facility care with 3-month refills	$2.51	$7.52	$10.03		$3.39 **
Community ART groups with 3-month refills	$0.99	$4.12	$5.12	-49.0%	
Community ART groups with 6-month refills	$0.99	$3.41	$4.40	-56.1%	

*Assumed full day for facility visit and ¼ day for DSD interaction at minimum wage for Lesotho. †Assumed half or full day at minimum wage for country, depending on total number of minutes reported by patients. ‡As reported by each study. **There is no minimum wage in Zimbabwe. Authors’ calculations based on 2021 “most typical annual salary” reported at https://www.averagesalarysurvey.com/zimbabwe. §Average across entire cohort. In both Malawi and Zambia2, only 23–46% of participants incurred any travel costs. Average cost/participant who did incur travel costs was thus substantially higher than is shown here.

## Discussion

In this synthesis of five previously reported studies, we pull together the average cost per patient treated for HIV and per patient retained in care for a variety of differentiated service delivery models in Lesotho, Malawi, Uganda, Zambia, and Zimbabwe. In most, but not all, cases, the DSD models achieved roughly the same 12-month retention rates as did conventional care or were reported as non-inferior to conventional care, a finding that likely reflects both the efficacy of the DSD models and the fact that most enrolled only stable patients who had already demonstrated their ability to adhere to treatment and remain in care. Some models did slightly better than conventional care in terms of retention; few did worse. We found that within countries, most models of care had relatively similar costs, except for resource-intensive models such as home ART delivery, which were more expensive, and, to a lesser extent, 6-month dispensing models, which were slightly less expensive.

We note that although provider costs varied widely among countries, from a low of $100 per patient per year in Zambia per year to a high of $187 per patient per year in Zimbabwe—both for conventional, facility-based care—these differences primarily reflect larger differences between countries in prices of inputs. Examples of such differences are provided in Table 5 and Table S1: the daily minimum wage in Malawi is $1.93, while in Lesotho it is $7.10, 3.8 times more; in Malawi, first-line ARVs averaged $6.30/month, while in Zimbabwe they cost $13.81/month, more than twice as much.

Unlike the differences among countries, the differences among models within countries are informative. The average cost per patient for each model reflects that model’s particular combination of location of service delivery, interaction frequency, provider cadre, and, in Uganda, proportions of patients on second-line regimens. Models that offered six-month dispensing, whether at the facility or in the community, consistently cost less than those with shorter dispensing durations, though the savings were modest in magnitude. The small differences in total cost/patient between models within countries highlight the large share of costs attributable to antiretroviral medications, whose cost does not vary with model of care. ARVs accounted for 60–92% of the total per patient, and laboratory tests (typically one viral load test/year) another 5–10%, on average. There is thus relatively little room for DSD models to reduce overall treatment costs.

For all the models, fidelity to model guidelines varied, with patients receiving more or fewer months of ARVs, viral load tests, and healthcare system interactions than guidelines recommended and often receiving different dispensing intervals than the model called for. These discrepancies, which were particularly noticeable in viral load test and clinic visit numbers (Table S2), affected the total resource utilization, and thus total cost, per patient. It is unclear whether strict fidelity to guideline recommendations is desirable for purposes of patient management, as clinical discretion may be preferable to guideline recommendations in some cases, and some patients may benefit from, for example, making more or fewer clinic visits than called for. In any case, increases in guideline compliance may cause costs either to rise or to fall, depending on the status quo.

The studies included in this synthesis differed from one another in several ways that are important to interpretation of their results. The models compared in the randomized trials are potentially substitutes for one another, enrolling the same populations in the same settings. In the two observational studies, in contrast, models should not be regarded as potential substitutes, because the settings, populations served, and other model characteristics varied widely by model. It is likely that a mix of models, including those that cost more and those that cost less than average, will be needed to provide access to a country’s entire ART patient cohort.

While most DSD models in most of the studies cost slightly less than conventional care, the models observed in Zambia1 were all somewhat more expensive than conventional care. This can largely be explained by the design of the models. In Zambia1, all four of the DSD models observed required more healthcare system interactions than did conventional care. These models were thus not “less intensive” than conventional care, and did not utilize fewer resources. In these models, resource allocation shifted from the clinic to the DSD model but did not diminish.

More striking than the cost implications to healthcare providers were the sharp reductions in costs to patients themselves. Patients reported cutting their own out-of-pocket and/or opportunity cost expenditures by between a quarter and a half per year, generally due to the reduced number of full clinic visits required by DSD models. Savings to patients may help improve long-term retention in care, as patient costs are often cited as a reason for interrupting treatment ^12^. Spending less time and money in seeking healthcare also provides an immediate improvement to patients’ quality of life.

Our study had a number of limitations. First, costing methods and resources included in the analysis differed between the studies, such that each “total cost per patient” estimate represents a slightly different set of inputs. Although we have noted inclusions and exclusions as fully as possible, the variation in input data and costing methods and the previously noted differences in national price levels argue for caution in comparing results between studies. Second, implementation of nearly all the models in the study was relatively recent, and costs may not reflect the operational efficiency that may be gained from experience. Third, observational studies in Uganda and Zambia were conducted prior to the scaleup of 6-month dispensing in these countries. Both countries now recommend 6-month dispensing, alone or in combination with other models, whenever possible, a development that is likely to reduce the costs of their models.

Fourth, we also are aware that the unit costs used for some categories of provider resources, such as labor and infrastructure, may not correctly capture the value of these resources to the health system. If a DSD model reduces the number of hours of a nurse’s time required per patient, for example, the cost estimates included here will reflect that as the product of the nurse’s salary and the estimated number of hours saved. From a health systems perspective, however, the value of that saved time will depend entirely on what the nurse does instead—be it seeing more patients, spending more time with the same number of patients, completing more administrative tasks, or taking longer breaks and working shorter hours. The provider must pay that nurse’s full-time salary regardless of how the “saved” time is spent. Effective management of human and physical resources is thus needed for DSD models to realize the apparent cost savings reported by these studies.

Finally, an important limitation to all of the studies reported here is that, for the most part, costs are limited to clients who are stable on ART and therefore eligible for DSD models. These clients are likely to cost the health system less than average even when in conventional care; shifting them to DSD models may leave the more expensive clients in conventional care, simply reallocating overall health system costs, rather than reducing them. Future research is needed to understand the role of DSD models in improving health outcomes and lowering per-patient costs of ART programmes as a whole. Research is also needed to explore the integration of DSD models for HIV treatment with service delivery for other chronic needs, to optimize clinic efficiency and minimize the overall burden of healthcare access on patients.

## Data availability

Figshare. Supplementary tables.docx. DOI: https://doi.org/10.6084/m9.figshare.17096678.v1 ^13^

Data are available under the terms of the Creative Commons Zero "No rights reserved" data waiver (CC BY 4.0 Public domain dedication).
