# Supplementary material for: Do differentiated service delivery models for HIV treatment in sub-Saharan Africa save money? Synthesis of evidence from field studies conducted in sub-Saharan Africa in 2017-2019
Source: Gates Open Res. 2022 Feb 25;5:177. Originally published 2021 Dec 8. [Version 2] doi: 10.12688/gatesopenres.13458.2 (PMC8907143; doi:10.12688/gatesopenres.13458.2)
Supplement: Peer review history

# Peer review: Reviewer response for version 2

**DOI:** 10.21956/gatesopenres.14845.r31814

**Published:** 2022-03-09

**Authors:** Michael J. Vinikoor

**Affiliations:** 1 Department of Medicine, University of Alabama at Birmingham, Birmingham, AL, USA; 2 Centre for Infectious Disease Research in Zambia, Lusaka, Zambia; 3 University of Zambia School of Medicine, Lusaka, Zambia

The authors have addressed my comments and concerns adequately.

Is the work clearly and accurately presented and does it cite the current literature?

Yes

If applicable, is the statistical analysis and its interpretation appropriate?

Yes

Are all the source data underlying the results available to ensure full reproducibility?

Yes

Is the study design appropriate and is the work technically sound?

Yes

Are the conclusions drawn adequately supported by the results?

Partly

Are sufficient details of methods and analysis provided to allow replication by others?

Partly

Reviewer Expertise:

infectious diseases, behavioral science, implementation science

I confirm that I have read this submission and believe that I have an appropriate level of expertise to confirm that it is of an acceptable scientific standard.

# Peer review: Reviewer response for version 1

**DOI:** 10.21956/gatesopenres.14715.r31567

**Published:** 2022-01-14

**Authors:** Michael J. Vinikoor

**Affiliations:** 1 Department of Medicine, University of Alabama at Birmingham, Birmingham, AL, USA; 2 Centre for Infectious Disease Research in Zambia, Lusaka, Zambia; 3 University of Zambia School of Medicine, Lusaka, Zambia

Summary:

 This article uses already published research on DSD models of HIV care across multiple Southern and East African countries to report on whether costs are reduced through this intervention that is now widely implemented. The justification for synthesizing these data could be better stated including the value of showing variability in cost savings across countries and by study design. The findings are limited by the fact that data came from research studies and not routine implementation by government systems, which will be using DSD for decades to come. Even in light of these limitations, this paper is significant in providing an informative view into the economics of DSD and would be useful to policymakers adopting and implementing DSD.

Background:

- In paragaph 1, the authors might want to add a sentence to state that the “conventional” model led to increasingly over-crowded clinics and this stretched staffing and led to queues, less patient-centered experiences with increased retention losses.
- Was saving cost one of the proposed outcomes of DSD? It would be good to state that it was never pitched as cost-saving or that in fact it was.
- Could you also reference that the term DSD could also be used to intensify services, such as for high HIV VL, advanced HIV, etc. Not all DSD should be for “less intensive” - I think it goes both ways (more and less intense, more and less costly) depending on need.
- In the last paragraph the authors could mention that DSD for stable patients was compared to convention model costs and outcomes for each site (assuming it was).
- Is this like a systematic review or meta analysis of published papers? How is this justified as the data are already published? Please explain further. Maybe you should mention that the cost outcomes and CE may differ across settings?

Methods:

- No ethics review: were all the data publically available via data sharing websites?
- Please comment on whether the costs of care before DSD/conventional approach were also collected as well as those from patients in conventional. Were those captured at the same time as DSD (to avoid calendar time biases)?
- Maybe having a column in Table 1 to indicate if the convention model was included in the data collection could be considered

Results:

- I note from Table 6, costs to patients, for several countries, moving from conventional care to 3 and then 6-month refills didn’t lead to a stepwise reduction in total costs. Why is that? In one case, Zambia, 3-month refills was more costly than conventional while 6-month was less. Please explain.

Discussion:

- As the DSD studies were implemented by NGOs, etc. and not by government, what are the implications of these findings? People often say that the government can find ways to do things cheaper than NGOs so perhaps we need additional data in the real world when the government is in charge of this.
- You comment that costs of DSD were highest in observational cohorts where second-line and people in first 6 months were included - didn’t this overall analysis focus on stable patients, which I though meant 1 ^st^ line and >6 months on ART? What is the meaning of this? Especially as trials (where you state the costs of DSD were more reduced) often don’t translate to the real world.
- It’s very interesting that most of the overall cost is due to ARVs and labs; hence DSD can have that much impact on cost. I suggest highlighting this more.
- You ought to clearly answer the question posed in the title in the first paragraph of the discussion. Do you consider DSD to save money?

Is the work clearly and accurately presented and does it cite the current literature?

Yes

If applicable, is the statistical analysis and its interpretation appropriate?

Yes

Are all the source data underlying the results available to ensure full reproducibility?

Yes

Is the study design appropriate and is the work technically sound?

Yes

Are the conclusions drawn adequately supported by the results?

Partly

Are sufficient details of methods and analysis provided to allow replication by others?

Partly

Reviewer Expertise:

infectious diseases, behavioral science, implementation science

I confirm that I have read this submission and believe that I have an appropriate level of expertise to confirm that it is of an acceptable scientific standard, however I have significant reservations, as outlined above.

# Response

**Published:** 2022-02-18

**Authors:** Sydney Rosen

We are glad that the Reviewer found our paper useful despite its limitations, which we also acknowledge.

*In paragaph 1, the authors might want to add a sentence to state that the “conventional”*

*model led to increasingly over-crowded clinics and this stretched staffing and led to queues, less patient-centered experiences with increased retention losses.*

 Thank you, a good suggestion. We have added this sentence.

*○ Was saving cost one of the proposed outcomes of DSD? It would be good to state that it was never pitched as cost-saving or that in fact it was.*

 Yes, it was. We have stated this in the first sentence of the second paragraph and provided a reference.

*○ Could you also reference that the term DSD could also be used to intensify services, such as for high HIV VL, advanced HIV, etc. Not all DSD should be for “less intensive” - I think it goes both ways (more and less intense, more and less costly) depending on need.*

 You are right and this is an important point. Almost all of the models in the papers we synthesized were intended to “less intensive,” with the exception of facility-based individual care in Uganda (which is for patients not eligible for less intensive models) and possibly of home and mobile ART delivery Zambia1. More intensive models designed for advanced HIV disease would not be expected to cost less. We have added a note about this to the first paragraph of the Introduction.

*○ In the last paragraph the authors could mention that DSD for stable patients was compared to convention model costs and outcomes for each site (assuming it was).*

 We assume that this refers to the last paragraph of the Introduction. We have added a note about this there.

*○ Is this like a systematic review or meta analysis of published papers? How is this justified as the data are already published? Please explain further. Maybe you should mention that the cost outcomes and CE may differ across settings?*

 We present it as a synthesis of published papers, rather than a systematic review or meta analysis, on the grounds that pulling together all the results of these papers into a single manuscript, where they are presented in a standard format and can be compared and contrasted, has value to readers. We have added a sentence about costs and outcomes varying across settings to the second to last paragraph of the introduction.

*Methods:*

*○ No ethics review: were all the data publically available via data sharing websites?*

 Yes, all the data used in the manuscript have either been published in open access journals or posted on an open access pre-print server. All are fully referenced in the manuscript.

*○ Please comment on whether the costs of care before DSD/conventional approach were also collected as well as those from patients in conventional. Were those captured at the same time as DSD (to avoid calendar time biases)?*

 While it would have been ideal to have data from prior to DSD implementation, to allow a pre-post or difference-in-differences comparison, all the papers were written after implementation of DSD models at the study sites. We sought retrospective or previously published data that would serve as a pre-period comparison but were unable to find it. All the models within each of the studies were observed at the same chronological time, to avoid calendar time biases. Table 1 indicates the dates of enrollment for each study, in case readers are concerned about secular changes underway during the study periods.

*○ Maybe having a column in Table 1 to indicate if the convention model was included in the data collection could be considered*

 In Table 2, a conventional care model is included and described for each study.

*Results:*

*○ I note from Table 6, costs to patients, for several countries, moving from conventional care*

*to 3 and then 6-month refills didn’t lead to a stepwise reduction in total costs. Why is that? In one case, Zambia, 3-month refills was more costly than conventional while 6-month was less. Please explain.*

 Good catch. We also noted that apparent discrepancy. As shown in Table 4, the number of visits made to the facility in Malawi and Zambia2 are roughly the same for conventional care and 3-month dispensing. What isn’t evident from Table 4 is that the time spent per visit was higher for 3-month dispensing than for conventional care in both Malawi and Zambia. This is reported in the original paper in Appendix 2 and evident in Table 6, where patient opportunity (time) costs for 3MD are larger than for conventional care in these two studies. We cannot explain why the visits were longer, unfortunately.

*Discussion:*

*○ As the DSD studies were implemented by NGOs, etc. and not by government, what are the*

*implications of these findings? People often say that the government can find ways to do things cheaper than NGOs so perhaps we need additional data in the real world when the government is in charge of this.*

 At this point in time, NGO partners with donor support, primarily but not solely from PEPFAR or the Global Fund, support a large number public sector healthcare facilities in much of sub-Saharan Africa. These sites are typically the countries’ highest-volume facilities and generally have better data collection than do sites without partner support, and new models of care are often initially introduced at these sites. As a result, these are the sites where research is done, particularly if the research is also donor-funded, as was the case for the studies included in this manuscript. While it’s likely that NGO partners increase costs in some cases, they do not do so in all. For example, for Malawi and Zambia2, the INTERVAL trial did not increase intervention costs beyond what would be required if no research partner or NGO were involved.

 Importantly, we cannot know that patient outcomes would be the same if the partners were not involved, and the logic of costing methodology suggests that all resources used to achieve an outcome should be included in cost estimates, including partner support. Based on the papers that were used to pull together these results, it appears that only the Uganda study attempted to capture any “above site” or partner expenses, and these were combined with infrastructure and fixed costs. These may well be equivalent to the costs that a government district health office would incur to supervise the implementation of DSD models in the district. For all these reasons, we’re not convinced that costs would be lower if implementation was done only by government, and we don’t have the data to make a case either way.

*○ You comment that costs of DSD were highest in observational cohorts where second-line and people in first 6 months were included – didn’t this overall analysis focus on stable patients, which I though meant 1 ^st^ line and >6 months on ART? What is the meaning of this? Especially as trials (where you state the costs of DSD were more reduced) often don’t translate to the real world.*

 This was a challenge in interpreting the observational studies. Unlike the trials, the observational studies included some DSD models that permitted patients on second line therapy and/or in their first 6 months on ART to enroll. We could not clearly exclude these patients in doing our synthesis. We have added a note about this to the limitations writeup in the Discussion. As you note, the trials, which could exclude all but stable patients, were not as close a reflection to real world conditions as were the observational studies.

*○ It’s very interesting that most of the overall cost is due to ARVs and labs; hence DSD can have that much impact on cost. I suggest highlighting this more.*

 (We assume you mean that “DSD can NOT have much impact on cost.”) Thank you for highlighting this point. It is important. We have emphasised it again in the Discussion where we contrast savings to providers with savings to patients.

*○ You ought to clearly answer the question posed in the title in the first paragraph of the discussion. Do you consider DSD to save money?*

 Good point! We have added an answer to this question in the last paragraph of the manuscript.

# Peer review: Reviewer response for version 1

**DOI:** 10.21956/gatesopenres.14715.r31568

**Published:** 2022-01-10

**Authors:** Ingrid Eshun-Wilson

**Affiliations:** 1 Division of Infectious Diseases,School of Medicine, Washington University, St. Louis, MO, USA

This important paper is an excellent comparison of costs of DSD models and standard care across multiple settings in sub-Saharan Africa, demonstrating overall comparable costs across all models and SOC, but with some reduced costs noted for 6MMD models, which highlights the importance of strengthening supply to allow 6M dispensations in several settings. The authors make a note of the importance of within country comparisons of models and provide extensive detail in very clear tables in the results section. Another critical finding is how guidelines for DSD model visit frequency do not necessarily match up with visit frequency in practice. The authors also highlight the key limitations of the analysis. The abstract can be strengthened to better articulate the main findings of the paper.

**I suggest accepting for indexing with minor revisions.**

**Abstract:**

 The results in the abstract are not entirely clear, this might be the result of the word count restrictions however, re-wording could aid understanding for the reader, currently the results of the study are hard to glean from the abstract alone and one must read the full paper to understand the abstract. I also recommend including a few more results in the abstract:

  

- It would be helpful if the authors could simplify/clarify the sentence “average provider cost/patient/year ranged from 100 -187 for conventional care, in both cases for facility-based conventional care”. Was this average cost assessed for conventional care only?

Consider simplifying and rephrasing to: “The average provider cost/patient/year for conventional care ranged from 100-187.” Although Table 3 suggests that this ranged from 86 to 187.

  

- I recommend re-wording: “Conventional care was less expensive than any other model in the Zambia observational study, more expensive than any other model in Lesotho, Malawi, and Zimbabwe, and in the middle of the range in the Zambia trial and the observational study in Uganda."

It is hard to determine what the synthesis is here, it is more a list of the results of individual studies, it would help the reader if the top-line points are made here.

 Consider a synthesized statement – e.g.: The majority of DSD models had comparable costs to SOC with the % difference in mean annual cost per patient ranging from 11.4% less to 9.2% more, with the exception of observational data from Zambia which showed much higher costs for DSD models compared to SOC (16-37% higher).

  

- I recommend rephrasing this sentence “Models incorporating 6-month dispensing were consistently less expensive to the provider per patient treated.” to include the comparison. Was this in comparison to all other models or compared to conventional care, compared to 3 month models? Based on reviewing the results in the paper one could rephrase as such: e.g. “Models incorporating 6-month dispensing were consistently less expensive to the provider per patient treated compared to all other models, including SOC (% difference range: -11.4% to -2.2%).”

This section is repeated in the main results of the paper and should be clarified in the main text results as well.

- The abstract conclusion could also be more specific by including a comparison statement.
  “In five field studies of the costs of DSD models for HIV treatment, most models within each country had relatively similar costs [to each other or conventional care or all], except for 6-month dispensing models, which were slightly less expensive.”
- The term “provider cost” throughout the abstract and manuscript is somewhat confusing as providers are frequently considered to be health providers in this setting rather than the health system/government/ngo, on reading the methods it is clear that it is related to most aspects of ART health service provision but possibly not staff salaries – consider changing it to “health service cost” or other term to avoid assumptions regarding what “provider” means.
- Ideally include the main study limitations in the abstract, particularly that what was included in unit cost estimates was quite variable across studies.

**Methods, Results & discussion:**

 Thank you for the overall clarity of the presentation of the methods and results.

- Lowest cost is cited as $100 in the abstract and conclusions, but from tables appears to be $86.
- Of note the cost of personnel which is a significant health care expenditure stands out as missing from the assessments in the main paper. Based on the reviewing the discussion section and supplementary materials, salaries were considered variably in the estimation of unit costs across studies and how they should be considered is unclear. It would however still be worthwhile being explicit and including in the main methods sections some detail about what a “clinic visit or off-site DSD visit” cost was comprised of and point out that salary may or may not have been included. This is in the footnotes of S1 Table, but I recommend mentioning briefly in main methods.
- In the patient costs section, it is worth clarifying why the total patient costs for Malawi and Zambia are higher for the 3-month models compared to SOC which is generally 1-2/3monthly. It appears from other tables that this may be related to more actual visits than in the guideline but this still doesn’t seem to account for the big % difference that is seen. It would be worth specifying in the text the specific reason, as ~50% savings to patients is one of the main conclusions of the paper.    

Is the work clearly and accurately presented and does it cite the current literature?

Yes

If applicable, is the statistical analysis and its interpretation appropriate?

Yes

Are all the source data underlying the results available to ensure full reproducibility?

Yes

Is the study design appropriate and is the work technically sound?

Yes

Are the conclusions drawn adequately supported by the results?

Yes

Are sufficient details of methods and analysis provided to allow replication by others?

Yes

Reviewer Expertise:

Infectious Diseases, Implementation Science, Systematic reviews, Epidemiology, Preference Elicitation, Mixed Methods

I confirm that I have read this submission and believe that I have an appropriate level of expertise to confirm that it is of an acceptable scientific standard.

# Response

**Published:** 2022-02-18

**Authors:** Sydney Rosen

We thank the reviewer for the positive overview.

*Abstract*

*The results in the abstract are not entirely clear, this might be the result of the word count restrictions however, re-wording could aid understanding for the reader, currently the results of the study are hard to glean from the abstract alone and one must read the full paper to understand the abstract.*

 We have attempted to clarify the abstract, in line with the suggestions below.

*I also recommend including a few more results in the abstract:*

*○ It would be helpful if the authors could simplify/clarify the sentence “average provider cost/patient/year ranged from 100 -187 for conventional care, in both cases for facility-*

*based conventional care”. Was this average cost assessed for conventional care only? Consider simplifying and rephrasing to: “The average provider cost/patient/year for conventional care ranged from 100-187.” Although Table 3 suggests that this ranged from 86 to 187.*

 We thank the reviewer for catching these discrepancies and have corrected these results in the abstract.

*○ I recommend re-wording: “Conventional care was less expensive than any other model in the Zambia observational study, more expensive than any other model in Lesotho, Malawi, and Zimbabwe, and in the middle of the range in the Zambia trial and the observational study in Uganda." It is hard to determine what the synthesis is here, it is more a list of the results of individual studies, it would help the reader if the top-line points are made here. Consider a synthesized statement – e.g.: The majority of DSD models had comparable costs to SOC with the % difference in mean annual cost per patient ranging from 11.4% less to 9.2% more, with the exception of observational data from Zambia which showed much higher costs for DSD models compared to SOC (16-37% higher).*

 We have accepted the reviewer’s suggestion for revising this sentence.

*○ I recommend rephrasing this sentence “Models incorporating 6-month dispensing were consistently less expensive to the provider per patient treated.” to include the comparison. Was this in comparison to all other models or compared to conventional care, compared to 3 month models? Based on reviewing the results in the paper one could rephrase as such: e.g. “Models incorporating 6-month dispensing were consistently less expensive to the provider per patient treated compared to all other models, including SOC (% difference range: -11.4% to -2.2%).”*

*This section is repeated in the main results of the paper and should be clarified in the main text results as well.*

 We have clarified this comparison in the abstract and the main text.

*○ The abstract conclusion could also be more specific by including a comparison statement. “In five field studies of the costs of DSD models for HIV treatment, most models within each country had relatively similar costs [to each other or conventional care or all], except for 6- month dispensing models, which were slightly less expensive.”*

 We have clarified this sentence in the abstract.

*○ The term “provider cost” throughout the abstract and manuscript is somewhat confusing as providers are frequently considered to be health providers in this setting rather than the health system/government/ngo, on reading the methods it is clear that it is related to most aspects of ART health service provision but possibly not staff salaries – consider changing it to “health service cost” or other term to avoid assumptions regarding what “provider” means.*

 “Provider cost” is a standard term in the health economics literature and is intended to distinguish costs to the healthcare system (the provider) from costs to patients themselves. We have added a definition to the methods section of the main text to address this. Space limitations prevent further explanation in the abstract.

*○ Ideally include the main study limitations in the abstract, particularly that what was included in unit cost estimates was quite variable across studies.*

 We have noted the most important limitation in the abstract. The limitation on abstract word count precludes providing any more detail.

*Methods, Results & discussion:*

*Thank you for the overall clarity of the presentation of the methods and results.*

*○ Lowest cost is cited as $100 in the abstract and conclusions, but from tables appears to be*

*$86.*

 As explained in the paragraph immediately after Table 4, we assumed that each Malawi cost/patient should be increased by $19 to reflect an average of one viral load test per year. Once $19 is added to each Malawi value, the lowest cost is then for conventional care in Zambia1, at $100/year. We have tried to clarify this in the text.

*○ Of note the cost of personnel which is a significant health care expenditure stands out asmissing from the assessments in the main paper. Based on the reviewing the discussion section and supplementary materials, salaries were considered variably in the estimation of unit costs across studies and how they should be considered is unclear. It would however still be worthwhile being explicit and including in the main methods sections some detail about what a “clinic visit or off-site DSD visit” cost was comprised of and point out that salary may or may not have been included. This is in the footnotes of S1 Table, but I recommend mentioning briefly in main methods.*

 Thank you for this suggestion. In fact, salaries, which as you note are an important contributor to cost, are included in all the estimates. We have added a sentence to explain this in the Methods section.

*○ In the patient costs section, it is worth clarifying why the total patient costs for Malawi and Zambia are higher for the 3-month models compared to SOC which is generally 1- 2/3monthly. It appears from other tables that this may be related to more actual visits than in the guideline but this still doesn’t seem to account for the big % difference that is seen. It would be worth specifying in the text the specific reason, as ~50% savings to patients is one of the main conclusions of the paper.*

 As shown in Table 4, the number of visits made to the facility in Malawi and Zambia2 are roughly the same for conventional care and 3-month dispensing. What isn’t evident from Table 4 is that the time spent per visit was higher for 3-month dispensing than for conventional care in both Malawi and Zambia. This is reported in the original paper in Appendix 2 and evident in Table 6, where patient opportunity (time) costs for 3MD are larger than for conventional care in these two studies.
